# The Nurse-Patient Relationship in Nursing Documentation: The Scope and Quality of Interactions and Prevalent Interventions in Inpatient Mental Health Units

**DOI:** 10.1155/2024/7392388

**Published:** 2024-05-29

**Authors:** Alonso Pérez-Toribio, Antonio R. Moreno-Poyato, María Teresa Lluch-Canut, Khadija El-Abidi, Gema Rubia-Ruiz, Ana María Rodríguez-López, Juan J. Pérez-Moreno, Marcelino Vicente Pastor-Bernabeu, Sara Sánchez-Balcells, Ana Ventosa-Ruiz, Montserrat Puig-Llobet, Juan F. Roldán-Merino

**Affiliations:** ^1^Mental Health Unit, Primary Care Service Delta de Llobregat/Primary Care Department, Costa de Ponent, Institut Català de la Salut, L'Hospitalet de Llobregat, Barcelona, Spain; ^2^Department of Public Health, Mental Health and Maternal and Child Health Nursing, Faculty of Nursing, Universitat de Barcelona, L´Hospitalet de Llobregat, Barcelona, Spain; ^3^Hospital Infanta Leonor, Madrid, Spain; ^4^University Hospital Center de Santiago, Santiago de Compostela, Spain; ^5^Psychiatry Service, Galdakao-Usansolo Hospital, Osakidetza-Basque Health Service, Galdakao-Usansolo, Spain; ^6^Department of Nursing at the Faculty of Health Sciences of the University of Alicante, Department of Health Alicante-Sant Joan D´Alacant, San Vicente del Raspeig, Spain; ^7^Campus Docent Sant Joan de Déu Fundació Privada, University of Barcelona, Barcelona, Spain

## Abstract

**Aims:**

(i) To evaluate the scope and quality of nurse-patient interactions recorded in the clinical notes of inpatient mental health units and (ii) to identify nursing interventions recorded in the context of the nurse-patient relationship in the clinical notes of inpatient mental health units.

**Design:**

A multimethod approach was use.

**Methods:**

Employing a quantitative cross-sectional design for the first aim, and a qualitative content analysis design of secondary data for the second aim. In total, 1,714 clinical notes were examined from 44 randomly selected patients who were hospitalized in five mental health units over the years 2022-2023.

**Results:**

The patient's experience of the interaction was present in 69.9% (*n* = 1,198) of the notes. However, only 12.0% (*n* = 205) of the notes reached a sufficient standard of quality in terms of describing the nurse-patient interactions. Specifically, more than half of the notes did not reflect any type of nursing intervention (*n* = 723; 60.4%). Thirty interventions compatible with the nursing intervention classification were identified, of which more than 70% corresponded to domains in the physiological area.

**Conclusion:**

This study shows that the quantity and scope of patients' clinical notes in mental health units do not sufficiently reflect the interventions performed by nurses, nor the quality or impact of these interventions in the context of the nurse-patient therapeutic relationship. *Implications for the Profession and/or Patient Care*. Improving the quality of clinical notes by integrating interventions and their impact can increase the quality of nursing care. *Impact*. The use of standardized nursing terminologies would contribute to the understanding of the extent and quality of nurse-patient interactions recorded in clinical notes. Thus, standardized documentation would also help to improve these interactions and their recording, which will facilitate decision-making. *Reporting Method*. Findings were reported using COREQ and STROBE guidelines. *Patient or Public Contributions*. There were no patient or public contributions.

## 1. Introduction

In the context of person-centered mental health care, proper recording of nursing documentation can be a key strategy for improving the quality and coordination of care and efficiency [1]. To this end, the patient's clinical documentation requires that nursing interventions and the quality of the nurse-patient relationship be properly recorded [2]. However, the literature points to significant deficiencies in documentation, particularly in acute mental health units, where the lack of detailed records affects the continuity and quality of care [3]. There is hardly any empirical evidence available on this issue at the international level, affecting clinical nursing practice in the field of mental health.

When patients are cared for in health services, especially within the hospital setting where they receive continuous care by different professionals, it is important that the care they receive is correctly recorded [4]. In nursing care, care interventions are recorded through nursing clinical notes, which collect a wide variety of information about the patient's care and progress [5]. Nursing clinical notes are defined as the record of nursing care that is planned and provided to patients by nurses or other caregivers with the nurse's supervision and approval [5]. Clinical notes are intended to show what happens in the nursing process and on what basis decisions are made during admission, together with interventions performed, nurse-patient rapport, progress assessments, and assessments by the patients themselves [6]. In addition, nursing clinical notes can be used for other purposes such as quality assurance, legal purposes, health planning, resource allocation, and nursing development and research. To achieve these purposes, nursing clinical notes should be structured [7], contain valid and reliable information, and comply with established standards [8]. Clear and concise nursing clinical notes help health care professionals to detect changes in patients' condition and improve the quality of care [9].

In particular, the recording of nursing interventions in clinical notes is an important part of nurses' work. The aim of these notes is to improve care by ensuring continuity of care [10, 11], enhancing patient safety, reducing miscommunication, and extending access to essential clinical information to all staff caring for a patient [12]. However, clinical notes are not always a description of what is actually done [13]. The quality of nursing clinical notes in some cases is inconsistent and often incomprehensible, poor, inaccurate, and inadequate regarding nursing care [14]. Sometimes, the effects of nursing interventions are neither visible nor verifiable [15]. The records tend to use terse language that is dominated by technical words, capture very limited aspects of nursing practice, and convey very little information to the lay reader [16].

In order to collect more accurate information from nursing notes, the literature recommends the use of standardized nursing terminologies (SNT) [13, 17, 18]. The use of SNTs can lead to improved decision-making, care effectiveness, and care plan evaluations [19], as well as creating links between nursing interventions and patient outcomes [7]. SNTs define nursing care, nursing interventions, and patient outcomes. SNTs can guide nurses through phases of the nursing process and can enhance the accurate formulation of patient care needs, planning of specific interventions, and communication of care [17]. SNTs have the potential to reduce variability in how nurses define their practice and document its impact on patient care [4]. The American Nurses' Association has recognized the nursing intervention classification (NIC) as the most widely used and best validated SNT classification, with good sustainability compared to other classifications for recording nursing interventions [17, 18]. It describes nursing interventions and consists of seven domains (physiological: basic, complex, behavioral, safety, family, health system, and community), designed to represent care provided in all settings and specialties [18]. In mental health, the most frequently addressed domains are safety and behavioral [19].

In the context of care in mental health units, nurses are by far professionals who interact the most with patients throughout the day [20]. Nursing interventions are placed within the framework of interaction with the patient through the therapeutic relationship [21]. From a person-centered care perspective, active collaboration between nursing staff and patients is necessary to identify personalized recovery goals and develop strategies to achieve those [22]. This collaboration hinges on the principles of attunement and bond, recognized as essential components of the therapeutic relationship [23]. To establish a therapeutic bond, communication marked by empathy, care, and warmth is imperative [24]. In the interplay between bonding and empathy lies the concept of empathic attunement, where practitioners fully engage with the client's internal perspective, acknowledging and adapting to their evolving experience [23]. Therefore, empathy and attunement are fundamental in nursing mental health interventions [25]. Attuning to someone involves sincere efforts to grasp their inner world and convey understanding, a cornerstone of the empathetic therapeutic relationship, recognized as a vital nursing skill for fostering bonds in mental health inpatient care [24, 26].

In this context, it is especially important to use the clinical notes for nurses to record what happens in the nurse-patient relationship [27]. Although interventions delivered in the context of the therapeutic relationship are known to be more effective and have an impact on patients' health outcomes [28], many interventions are not recorded in the course of clinical care [27, 29, 30], rather the records focus more on the problems and symptoms presented by patients [16]. Although previous research has demonstrated that the quality of mental health nursing documentation needed to be improved to ensure continuity and quality of patient care [31], the lack of quality indicators poses a challenge for the search for high quality nursing documentation [17]. Along these lines, a study conducted by Myklebust and Bjørkly, 2019 in Norway, concluded that only 7.6% of the excerpts from the progress notes sufficiently described the interactions in terms of the rapport process in the nurse-patient relationship [27].

Despite the importance of the nurse-patient therapeutic relationship in mental health care, the literature highlights the inconsistency and lack of detail of nursing interventions reflected in clinical notes. These deficiencies compromise the continuity and quality of care, affecting clinical decision-making and, ultimately, patient health outcomes [1, 3]. It seems necessary to fill the existing gap in empirical evidence on the quality and extent of nursing documentation in mental health by providing concrete data on the quality of records and the nature of interventions.

The present study was designed with a dual purpose: (i) to evaluate the scope and quality of nurse-patient interactions recorded in the clinical notes of inpatient mental health units and (ii) to identify nursing interventions recorded in the context of the nurse-patient relationship in the clinical notes of inpatient mental health units.

## 2. Materials and Methods

### 2.1. Study Design

A multimethod approach was used for the present study. A quantitative cross-sectional design was used to assess the extent and quality of the nurse-patient interactions recorded in the clinical notes. Also, a qualitative content analysis design of secondary data in clinical notes was used to identify the nursing interventions recorded in the context of the nurse-patient relationship.

### 2.2. Sample and Setting

The sample consisted of clinical notes from patients who were hospitalized in mental health units in Spain during the years 2022-2023. Five units were randomly selected for this study which was part of a multicenter project of 12 hospitals of the national health system in Spain called RTS_MHNursing. In this project, all the units were closed units for the care of adults with acute mental health problems. They represented the different geographic areas of the Spanish territory.

The patients included in the study were selected consecutively in each of the units chosen according to the same inclusion criteria as in the larger project. Therefore, adult patients, who consented to participate in the study on a voluntary basis, hospitalized in mental health inpatient units. Participants were excluded if at the time of recruitment they presented a language barrier, mechanical restraint, contraindication by the clinical referent, cognitive impairment, or intellectual disability. No other exclusions were made to maximize the external validity of the study. For the calculation of the sample size of clinical notes required, a study with the same objective, design, and evaluation instrument was used as a reference [25]; thus, the final sample consisted of a total of 1,714 nursing clinical notes from 44 patients who had been hospitalized in 5 mental health units in Spain during the years 2022-2023.

### 2.3. Data Collection

The researcher responsible for each unit was in charge of collecting the data from the patients' clinical documentation. She was responsible for anonymizing the names of the professionals and names of the patients in the nursing clinical notes of each of the patients included in the study. Records of professionals other than nurses were removed from the data before they were analyzed. The study data were collected and managed using REDCap electronic data capture tools [32]. In accordance with the research objectives, clinical notes in which the nurse and patient did not have an opportunity to interact were excluded. If a clinical note contained more than one episode of interaction and different patient experiences were reported in relation to these episodes, the note was divided and counted as a separate note.

### 2.4. Measurement Instrument

To evaluate the extent and quality of nurse-patient interactions recorded, we used the Spanish version of the scale for the evaluation of staff-patient interactions in progress notes [6] and applied it to the selected clinical notes. The SESPI-SP scale assesses the quality of nurse-patient interactions recorded in the notes through patients' experience and the interventions made by the nurses and whether this intervention succeeds in meeting the emotional needs of the patients. This scale consists of four steps detailed as follows:

1. Step 1 aims to know if the patient's experience is described in the clinical note, “how is the patient when interacting with the nurse.” This is answered dichotomously (Yes/No), and only an affirmative answer enables one to move to the next step.
2. Step 2 evaluates whether the patient's experience within the interaction with the nurse was positive or negative. Depending on the degree of satisfaction or dissatisfaction with the patient's response, a response of –II, –I, +I, or +II is given.
3. In step 3, one must identify and categorize the nurse's response to the interaction described in the clinical note. Four possible responses are found: (a) the nurse's response is not described in the clinical note; (b) the nurse's response is known, but not the patient's reaction; (c) the nurse's response and the patient's reaction is known, but not the patient's feelings; (d) the nurse's response, the patient's reaction, and the feelings produced by this intervention are recorded, and this feeling with the nurse is called attunement. Only this response enables one to move on to the next step.
4. In step 4, this attunement is evaluated positively or negatively depending on whether it has failed or succeeded. Again, the answer is –II, –I, +I, or +II.

## 3. Data Analysis

To analyze the quantitative data, descriptive statistics were calculated using the IBM SPSSv27 program. The results were presented in the form of frequency and percentages. The qualitative data analysis procedure was based on content analysis [33] and nurse interventions were identified from free-text nursing records in the acute mental health unit setting. First, the first author read the entire text to become familiar with the data. Because the text was not standardized, a data extraction matrix was designed to aid with a proper follow-up. Interventions were then inductively identified through a systematic mapping process. Descriptions of nurses' actions were then categorized using a deductive approach that reflected the NIC definition of nursing interventions. These descriptions revealed the interventions that nurses performed for or with patients in the context of patient interactions. The first author repeated this analysis process after one month and compared their identifications of NIC interventions by revisiting the original texts and with the rest of the research team.

### 3.1. Ethical Considerations

This study was approved by the Ethical Committees of five hospitals, and research permission was granted. The data were analyzed anonymously to preserve the anonymity of patients and staff.

## 4. Results

### 4.1. Scope and Quality of Interactions in Clinical Notes

**Figure 1:**
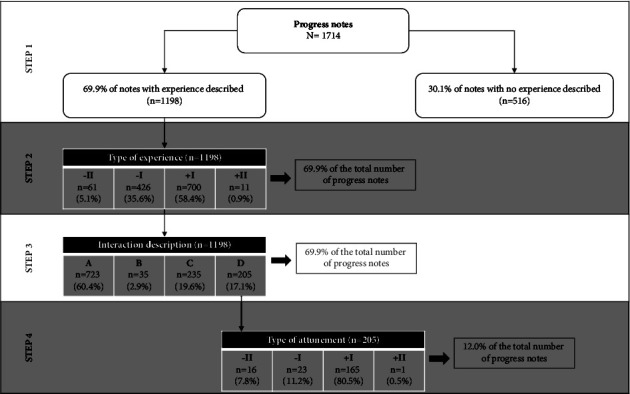
Distribution of the scope and quality of interactions in clinical notes.

A total of 1,714 clinical notes were analyzed.  Figure 1 provides an overview of the distribution of clinical notes according to four stages of classification as the quality of records increases. The most remarkable result was that only 12.0% (*n* = 205) of notes described the interactions in a sufficient detail to be fully analyzed in terms of recording attunement.

### 4.2. Description of Patient Experiences in Clinical Notes

Although the description of the patient experience was present in 69.9% (*n* = 1,198), almost one-third of the clinical notes did not include any patient-related experience. In most cases, nurses recorded positive and negative patient experiences in the middle range; only 8.3% of notes reflected an extreme positive or negative patient experience.

### 4.3. Description of Nurse-Patient Interactions in Clinical Notes

Of the notes in which the patient's experience of the interaction had been described, in more than half, no nurse intervention of any kind was recorded (*n* = 723; 60.4%). In only 17.1% (*n* = 205) of the cases, both nurse interventions and patient responses to that intervention were described. Only 205 of the 1,714 notes (12%) could be evaluated in relation to the quality of the interaction. In 7.8% of the cases, there was a connection in the failed interaction (−II), 0.5% were in the + II or successful connection category, and the majority of interactions were classified as partially failed (11.2%) and partially successful (80.5%).

### 4.4. Nursing Interventions Identified in Clinical Notes

Of the 1,714 notes analyzed, almost two thirds (*n* = 1250; 72.9%) were found to contain an intervention compatible with the NIC taxonomy. From the data analysis, 30 NIC interventions were identified. Nearly 2/3 of the interventions recorded by nurses in the clinical notes were classified as “2304-medication administration: oral” (62.6%), followed by “5360-entertainment therapy” (10.4%). Other prevalent interventions were “4920-active listening” present in 6.1% of the occasions and “5270-emotional support,” present in 4.3% of clinical notes (Table 1). It should be noted that more than 70% of interventions recorded corresponded to domains in the physiological area. The total number of interventions identified and their distribution can be found in Supplementary File 1.

## 5. Discussion

The present study had a dual purpose; first, to assess the extent and quality of nurse-patient interactions recorded in clinical notes, and second, to identify nursing interventions recorded in the context of the nurse-patient relationship in clinical notes. Regarding the first purpose, the results show that only one out of 10 notes written by nurses sufficiently described interactions to enable a full analysis in terms of the quality of the recording of the nurse-patient interaction process. The most noteworthy result of the second purpose was that most of the nursing interventions recorded in the notes in the context of the nurse-patient relationship respond to interventions of the physiological domain, rather than interventions of a more relational nature, as could be expected in the context of nursing care in acute mental health units.

Nursing clinical notes should reflect how staff understand their patients, how they interact and what nursing care they receive [9]. Deacon and Fairhurst [34] described nursing care in psychiatric hospitalization as a set of activities that are hardly visible and difficult to conceptualize, characterized by individual attention, as well as by routine and continuous work.

Our study, a finding which is compatible with the study by Myklebust et al., showed that 1/3 of total nursing clinical notes studied contained no description of patient experiences [14]. However, the most notable finding was that only 12% (*n* = 205) of extracts described nurse-patient interactions to an extent that enabled them to be analyzed in terms of quality. This means that up to 88% of extracts were impossible to evaluate according to attunement, which is a fundamental characteristic of the therapeutic relationship. Therefore, in most of the notes, the quality of the bond, an essential characteristic in the nurse-patient therapeutic relationship, could not be assessed [24]. These results are in line with previous research that has found that traditionally, nurses underestimate the value of their documentation and the extent to which their diagnostic accuracy leads to better patient outcomes [4].

In most cases, in clinical notes, nurses recorded descriptions of patients' experiences in terms of positive and negative attunement distributed in a medium range; thus, only 8.3% of notes reflected an extreme positive or negative experience of the patients. This result is in complete agreement with the findings by Myklebust and Bjørkly [25].

In over half of clinical notes, no intervention of any kind was recorded by the nurse towards the patient once there had been an interaction. Myklebust et al. [14] concluded that while writing clinical notes, nurses position themselves as observers, and although the relationship between staff and patients is essential, nurses do not consider the relevance of documenting these interactions. This may be one reason why the clinical notes included very little information about what the nurses had done to help the patient, other than observing and coordinating care.

Only one in five of clinical notes that did record the patient-related experience described both the nurses' interventions and the patients' responses. As in other studies, our findings indicate that nursing documentation does not accurately describe actual nursing care [27, 29, 30].

Inpatients positively value the ability of health care personnel to understand and comprehend their experiences and feelings [24]. This may explain why clinical notes recording the interventions and the nurse-patient attunement in the interactions between them are mostly partially satisfactory (80.5%).

The second objective of our study was to identify and describe nursing interventions on nurse-patient interactions in clinical notes of acute mental health units using the NIC domains as a framework for assessing the outcomes of nursing clinical notes. In our study, almost two thirds of the nursing notes studied showed some intervention compatible with the NIC taxonomy. Frauenfelder et al. demonstrated that the NIC covers the essential nursing care interventions, since 89.4% of all the intervention descriptions in their study fully coincided with the NIC [19]. However, our study is close to the one conducted by De Groot et al. [3] which concluded that only half of the respondents used SNT in their clinical notes. Ameel et al. [15] concluded that interventions such as presence or listening may not be recorded, as they are not perceived as interventions because they are an obvious part of nursing care.

In our study, the “active listening” intervention hardly appears; this finding may be because the nurses consider this intervention to be routine and for this reason, they do not consider it necessary in recording this intervention.

Another study conducted by Frauenfelder et al. [19] showed that the NIC intervention “surveillance” was essential as they concluded that the need for surveillance is one of the reasons for admitting patients to acute inpatient facilities for nurses to assess patients through interactions and observation of symptoms. However, in our study this intervention is not collected, only 30 NIC interventions were identified, a result far removed from the one performed by Ameel et al. [15]; who identified 71 different nursing interventions, 64 of which are described in the NIC. This lack of collection and recording of the interventions performed by the nurses in our study can be understood according to the findings by Ameel et al. [15] and Fore et al. [29] who explain that this occurs when nurses have integrated interventions as part of their work and do not record them.

In our study most of the interventions recorded corresponded to domains in the physiological area, represented by the administration of both oral and intramuscular medication and wound care. If we compare the results of our study with others conducted in a similar setting, hospital care. Ameel et al. [15] and Fraunfelder et al. [19] most prevalent interventions were in the safety and behavioral domains. The safety domain is not represented among the most prevalent NICs in our study, even though vigilance is one of the tasks most frequently performed in acute mental health units. This result can be explained by the delegation of competencies in the interdisciplinary team. However, the behavioral domain appears to be represented in several interventions such as (limit setting 4380; recreation therapy 5360; active listening 4920; emotional support 5270; behavior modification 4360; anxiety reduction 5820; conflict mediation 5020; anger control assistance 4640). Another study conducted by Thomé et al. [35] analyzed the documentation of patients in an outpatient center in Brazil, and their most prevalent interventions were assistance in self-care, socialization, and promotion of exercise. In this case, these interventions have not been collected in our study of clinical notes. This difference may be explained by the different health care context.

Nurses have a deep-rooted biomedical model of care [28], which influences the therapeutic relationship [14]. Therefore, these findings could in part explain the incomplete or inadequate nursing records in patients' clinical notes, or records based more on the biomedical model than on nurse-patient interactions [30].

## 6. Strengths and Limitations

The nursing clinical notes from the selected facilities did not use SNT, which means that results are based on the analysis of free-text notes, which included very few direct descriptions of nursing interventions. With free-text note analysis, there is a possibility of using too much interpretation during the process of analyzing the notes.

Due to the large number of randomized extracts and the fact that around 100 people wrote in the selection period, the risk of author bias was low in the dataset.

One of the strengths of the present study was the considerable number of randomized evolution notes collected from five different acute mental health units. The five mental health units chosen for this research represented a wide range of patient illness stages, diagnoses, and degrees of coercive or voluntary treatment.

## 7. Implications for Nursing Management

To our knowledge, there are few international quantitative studies measuring the quantity and quality of staff-patient interactions in nursing documentation in mental health settings. Consequently, the present study appears to represent an important step in the effort to explore the extent and quality of documented staff-patient interactions and the quality of documented nurse-patient interactions.

Improving the quality of clinical notes by integrating interventions and their impact, also considering the degree of nurse-patient harmony, can contribute to an increase in the quality of nursing care. This could increase patient satisfaction with the admission and their experience. It would be interesting to try to raise awareness of the importance of a systematized record of nursing clinical courses that include SNT and patient experiences related to the interventions, which would reinforce the training curriculum of mental health nurses.

The SESPI-Sp can provide data to qualitatively assess nursing documentation in relation to nursing interventions performed in the context of interactions with patients in mental health units. In this manner, it contributes to the improvement of the quality of mental health nursing care.

The SNT could play an important role in the development of nursing documentation, thereby becoming a better source for the evaluation and development of staff-patient interactions in clinical practice.

A more standardized use would contribute to the understanding of the extent and quality of nurse-patient interactions recorded in clinical notes. In this manner, standardized documentation would also help to improve these interactions and their recording, which will facilitate decision-making and help to provide solutions to problems that may arise during patient care.

Reading the clinical notes log explored in this study suggests that nurses record very little of the time they spend interacting with patients in the context of the therapeutic relationship. These insights highlight the need for interventions to establish and develop the nurse-patient relationship. Consequently, it could be of interest to managers when planning the structure and staffing needs in inpatient mental health care.

## 8. Conclusions

The results of this study provide insight into the quality and quantity of nursing clinical notes related to patient experiences and nurse-patient interactions. Of the total number of clinical notes studied, only 12% described the relationship and rapport between nurse and patient in their interactions with sufficient quality. This means that the patients' feelings and experiences revealed in the context of the nurse-patient relationship are not adequately recorded in the documentation.

The clinical notes studied identified the recorded nursing interventions, 30 of which are described in the NIC. However, interventions of a relational nature, which are considered highly relevant and important in mental health nursing care, were almost nonexistent in the clinical documentation.

Consequently, nursing clinical notes did not fully and adequately record the interventions that nurses perform, as well as the impact of the interventions.

## Figures and Tables

**Table 1:** Distribution of identified nursing interventions' classification and related domains.

Nursing interventions' classification	*n* (%)	Domains
2304-medication administration: oral	783 (62.6)	Physiological: complex
5360-recreation therapy	130 (10.4)	Behavioral
4920-active listening	76 (6.1)	Behavioral
5270-emotional support	54 (4.3)	Behavioral
2313-medication administration: intramuscular (IM)	30 (2.4)	Physiological: complex
4380-limit setting	25 (2)	Behavioral
6580-physical restraint	18 (1.4)	Physiological: basic
1800-self-care assistance	16 (1.3)	Physiological: basic
3660-wound care	16 (1.3)	Physiological: complex
4360-behavior modification	16 (1.3)	Behavioral
5820-anxiety reduction	14 (1.1)	Behavioral
5020-conflict mediation	12 (1)	Behavioral
4640-anger control assistance	10 (0.8)	Behavioral
Others	50 (4)	

*n* = 1250.

## Data Availability

The data that support the findings of this study are available from the corresponding author upon reasonable request.

## Supplementary Materials

Supplementary Materials Supplementary File 1: Total number of interventions identified in the clinical notes studied and their distribution by frequency and percentage.
