# Supplementary material for: The Nurse-Patient Relationship in Nursing Documentation: The Scope and Quality of Interactions and Prevalent Interventions in Inpatient Mental Health Units
Source: J Nurs Manag. 2024 May 29;2024:7392388. doi: 10.1155/2024/7392388 (PMC11918906; doi:10.1155/2024/7392388)
Supplement: Supplementary Materials — Supplementary File 1: Total number of interventions identified in the clinical notes studied and their distribution by frequency and percentage. [file 7392388.f1.docx]

**TITLE**

The nurse-patient relationship in nursing documentation: the scope and quality of interactions and prevalent interventions in inpatient mental health units

**The full names of the authors-**

1. Alonso PÉREZ-TORIBIO
2. Antonio R. MORENO-POYATO
3. María Teresa LLUCH-CANUT
4. Khadija EL-ABIDI
5. Gema RUBIA-RUIZ
6. Ana María RODRÍGUEZ-LÓPEZ
7. Juan J. PÉREZ-MORENO
8. Marcelino Vicente PASTOR-BERNABEU
9. Sara SÁNCHEZ-BALCELLS
10. Ana VENTOSA-RUIZ
11. Montserrat PUIG-LLOBET
12. Juan F. ROLDÁN-MERINO

**The author's institutional affiliations at which the work was carried out**

1. Alonso Pérez-Toribio

Mental Health Unit, Primary Care Service Delta de Llobregat / Primary Care Department. Costa de Ponent. Institut Català de la Salut. L'Hospitalet de Llobregat, Barcelona, Spain

[alonperez11@gmail.com](mailto:alonperez11@gmail.com)

1. Antonio R. Moreno-Poyato **CorrespondingAuthor**

Department of Public Health, Mental Health and Maternal and Child Health Nursing, Faculty of Nursing, Universitat de Barcelona, L´Hospitalet de Llobregat, Spain

Research Group in Mental Health Nursing Care – NURSEARCH

[amorenopoyato@ub.edu](mailto:amorenopoyato@ub.edu)

1. María Teresa Lluch-Canut

Department of Public Health, Mental Health and Maternal and Child Health Nursing, Faculty of Nursing, Universitat de Barcelona, L´Hospitalet de Llobregat, Spain

Research Group in Mental Health Nursing Care – NURSEARCH

[tlluch@ub.edu](mailto:tlluch@ub.edu)

1. Khadija EL-Abidi

Department of Public Health, Mental Health and Maternal and Child Health Nursing, Faculty of Nursing, Universitat de Barcelona, L´Hospitalet de Llobregat, Spain

Institut de Neuropisquiatria i Addiccions, Centre Fòrum, Hospital del Mar. Barcelona, Spain.

[kadi.elabidi@ub.edu](mailto:kadi.elabidi@ub.edu)

1. Gema Rubia-Ruiz

Hospital Infanta Leonor, Madrid, Spain

[gema.rubia@salud.madrid.org](mailto:gema.rubia@salud.madrid.org)

1. Ana María Rodríguez-López

University Hospital Center de Santiago, Santiago de Compostela, España

[Ana.Maria.Rodriguez.Lopez@sergas.es](mailto:Ana.Maria.Rodriguez.Lopez@sergas.es)

1. Juan J. Pérez-Moreno

Psychiatry Service, Galdakao-Usansolo Hospital, Osakidetza-Basque Health Service, Galdakao-Usansolo, Spain

[juanjose.perezmoreno@osakidetza.eus](mailto:juanjose.perezmoreno@osakidetza.eus)

1. Marcelino Vicente Pastor-Bernabeu

Department of Nursing at the Faculty of Health Sciences of the University of Alicante

Department of health Alicante-Sant Joan d´Alacant

[pastor_mvi@gva.es](mailto:pastor_mvi@gva.es)

1. Sara Sánchez-Balcells

Department of Public Health, Mental Health and Maternal and Child Health Nursing, Faculty of Nursing, Universitat de Barcelona, L´Hospitalet de Llobregat, Spain

Research Group in Mental Health Nursing Care – NURSEARCH

[sara.sanchez@ub.edu](mailto:sara.sanchez@ub.edu)

1. Ana Ventosa-Ruiz

Department of Public Health, Mental Health and Maternal and Child Health Nursing, Faculty of Nursing, Universitat de Barcelona, L´Hospitalet de Llobregat, Spain

Benito Menni Mental Health Service Center, Hospitalet de Llobregat, Barcelona

Research Group in Mental Health Nursing Care – NURSEARCH

[anaventosaruiz@ub.edu](mailto:anaventosaruiz@ub.edu)

1. Montserrat Puig-Llobet

Department of Public Health, Mental Health and Maternal and Child Health Nursing, Faculty of Nursing, Universitat de Barcelona, L´Hospitalet de Llobregat, Spain

Research Group in Mental Health Nursing Care – NURSEARCH

[monpuigllob@ub.edu](mailto:monpuigllob@ub.edu)

1. Juan F. Roldán-Merino

Campus Docent Sant Joan de Déu Fundació Privada. University of Barcelona, Spain

Research Group in Mental Health Nursing Care – NURSEARCH

[jroldan@santjoandedeu.edu.es](mailto:jroldan@santjoandedeu.edu.es)

**Total number of interventions identified in the clinical notes studied and their distribution by frequency and percentage**

| **NIC** | | | |
| --- | --- | --- | --- |
|  | **Frequency** | **Percentage** | **Valid percentage** |
| **Total** | 1250 | 72,9 | 100,0 |
| 2304- Medication Administration: Oral | 783 | 45,7 | 62,6 |
| 5360- Recreation Therapy | 130 | 7,6 | 10,4 |
| 4920- Active Listening | 76 | 4,4 | 6,1 |
| 5270- Emotional Support | 54 | 3,2 | 4,3 |
| 2313- Medication Administration: Intramuscular (IM) | 30 | 1,8 | 2,4 |
| 4380- Limit Setting | 25 | 1,5 | 2,0 |
| 6580- Physical Restraint | 18 | 1,1 | 1,4 |
| 1800- Self-Care Assistance | 16 | 0,9 | 1,3 |
| 3660- Wound Care | 16 | 0,9 | 1,3 |
| 4360- Behavior Modification | 16 | 0,9 | 1,3 |
| 5820- Anxiety Reduction | 14 | 0,8 | 1,1 |
| 5020- Conflict Mediation | 12 | 0,7 | 1,0 |
| 4640- Anger Control Assistance | 10 | 0,6 | 0,8 |
| 6630- Seclusion | 8 | 0,5 | 0,6 |
| 6680- Vital Signs Monitoring | 6 | 0,4 | 0,5 |
| 6510- Hallucination Management | 5 | 0,3 | 0,4 |
| 6160- Crisis Intervention | 4 | 0,2 | 0,3 |
| 2395- Medication Reconciliation | 3 | 0,2 | 0,2 |
| 4354- Behavior Management: Self-Harm | 3 | 0,2 | 0,2 |
| 5880- Calming Technique | 3 | 0,2 | 0,2 |
| 6610- Risk Identification | 3 | 0,2 | 0,2 |
| 1850- Sleep Enhancement | 2 | 0,1 | 0,2 |
| 3786- Treatmenthyperthermia | 2 | 0,1 | 0,2 |
| 4035- Capillary Blood Sample | 2 | 0,1 | 0,2 |
| 6450- Delusion Management | 2 | 0,1 | 0,2 |
| 6487- Environmental Management: Violence Prevention | 2 | 0,1 | 0,2 |
| 6654- Surveillance: Safety | 2 | 0,1 | 0,2 |
| 0612- Urinary Incontinence Care: Enuresis | 1 | 0,1 | 0,1 |
| 6340- Suicide Prevention | 1 | 0,1 | 0,1 |
| 6658- Surveillance: Remote Electronic | 1 | 0,1 | 0,1 |
| Total | 1250 | 72,9 | 100,0 |
| System | 464 | 27,1 |  |
|  | 1714 | 100,0 |  |
